# Supplementary figures and images for: The development of extracellular vesicle markers for the fungal phytopathogen Colletotrichum higginsianum
Source: J Extracell Vesicles. 2022 May 6;11(5):e12216. doi: 10.1002/jev2.12216 (PMC9077143; doi:10.1002/jev2.12216)

(a)

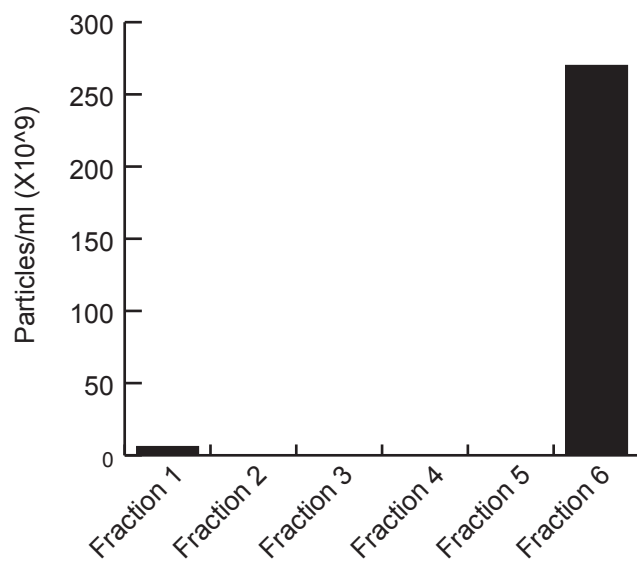

(b)

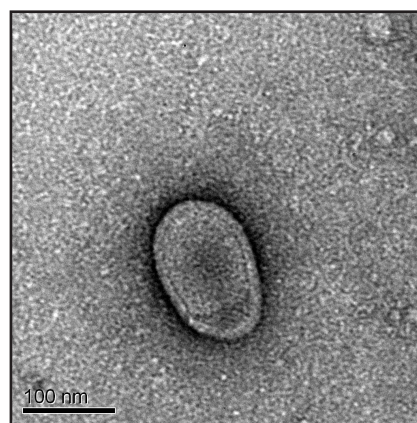

Supplement: Supplementary file 1 — FIGURE S1: Preliminary attempts at isolating EVs from the supernatant of C. higginsianum mycelia. Supernatant from a mycelial liquid culture was processed for EVs. Crude vesicle pellets were bottom‐loaded into a discontinuous Optiprep gradient consisting of 5, 10, 20 and 40% layers. After centrifugation at 100K x g for 17 h, the 5% layer was discarded and the next six fractions of 1 ml each were collected and processed with further ultracentrifugation. (a) Nanoparticle tracking (NTA) data showing the average concentration of particles in each of the collected Optiprep fractions. (b) TEM image of an EV‐like particle found in fraction 6. [file JEV2-11-e12216-s003.pdf]

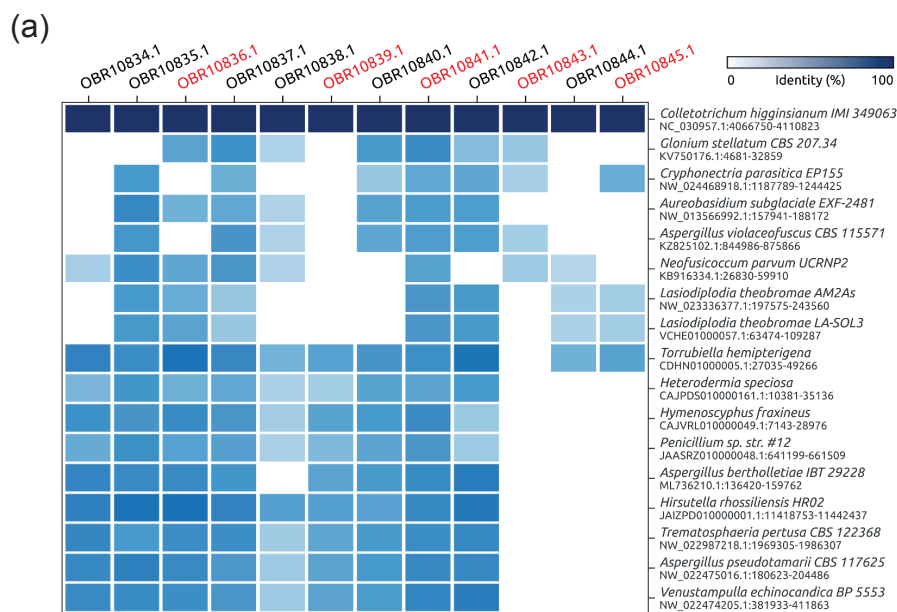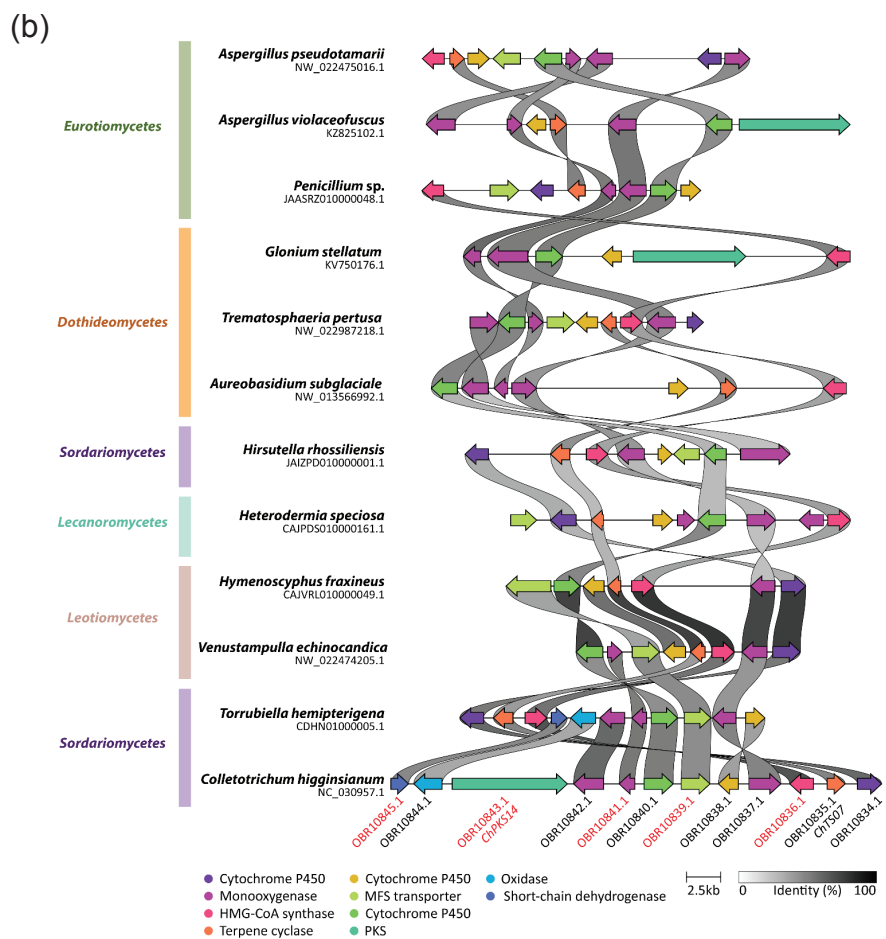

Supplement: Supplementary file 2 — FIGURE S2: Homologous biosynthetic gene clusters of BGC21 from C. higginsianum. (a) Heatmap of the hits detected using cblaster v1.3.9 (Gilchrist et al., 2021) with default parameters and (b) rearrangement of BGCs depicted using clinker v0.0.21 (Gilchrist et al., 2021). IDs in red correspond to proteins detected in at least two biological replicates of EVs preparations. [file JEV2-11-e12216-s001.pdf]

(a)

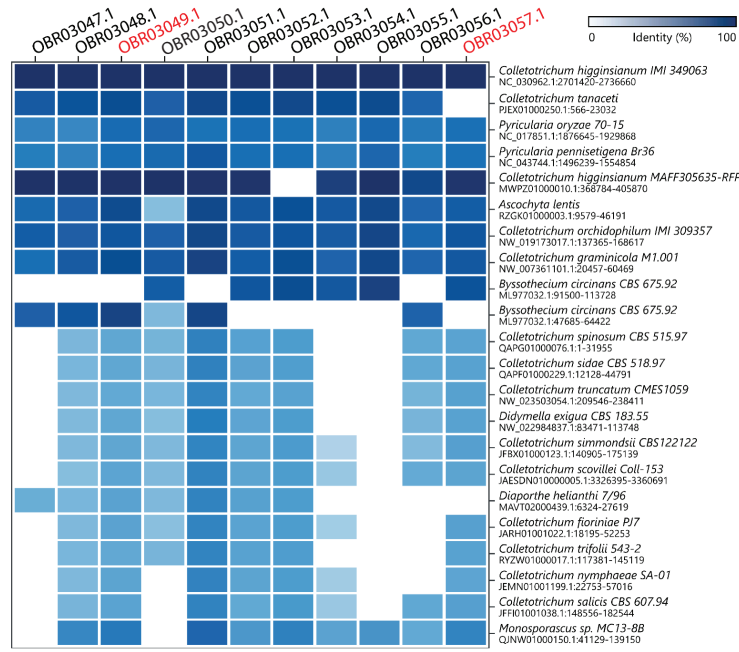

(b)

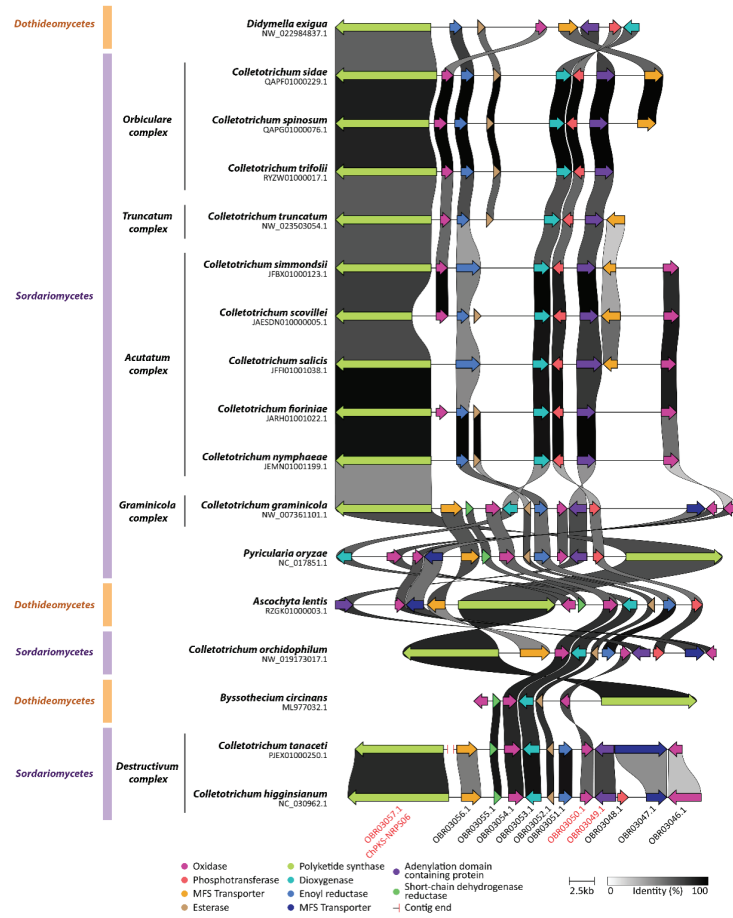

Supplement: Supplementary file 3 — FIGURE S3: Homologous biosynthetic gene clusters of BGC71 from C. higginsianum. (a) Heatmap of the hits were detected using cblaster v1.3.9 (Gilchrist et al., 2021) with default parameters and (b) rearrangement of BGCs depicted using clinker v0.0.21 (Gilchrist et al., 2021). IDs in red correspond to proteins detected in at least two biological replicates of EVs preparations. Species complexes were indicated where appropriate for Colletotrichum spp. [file JEV2-11-e12216-s006.pdf]

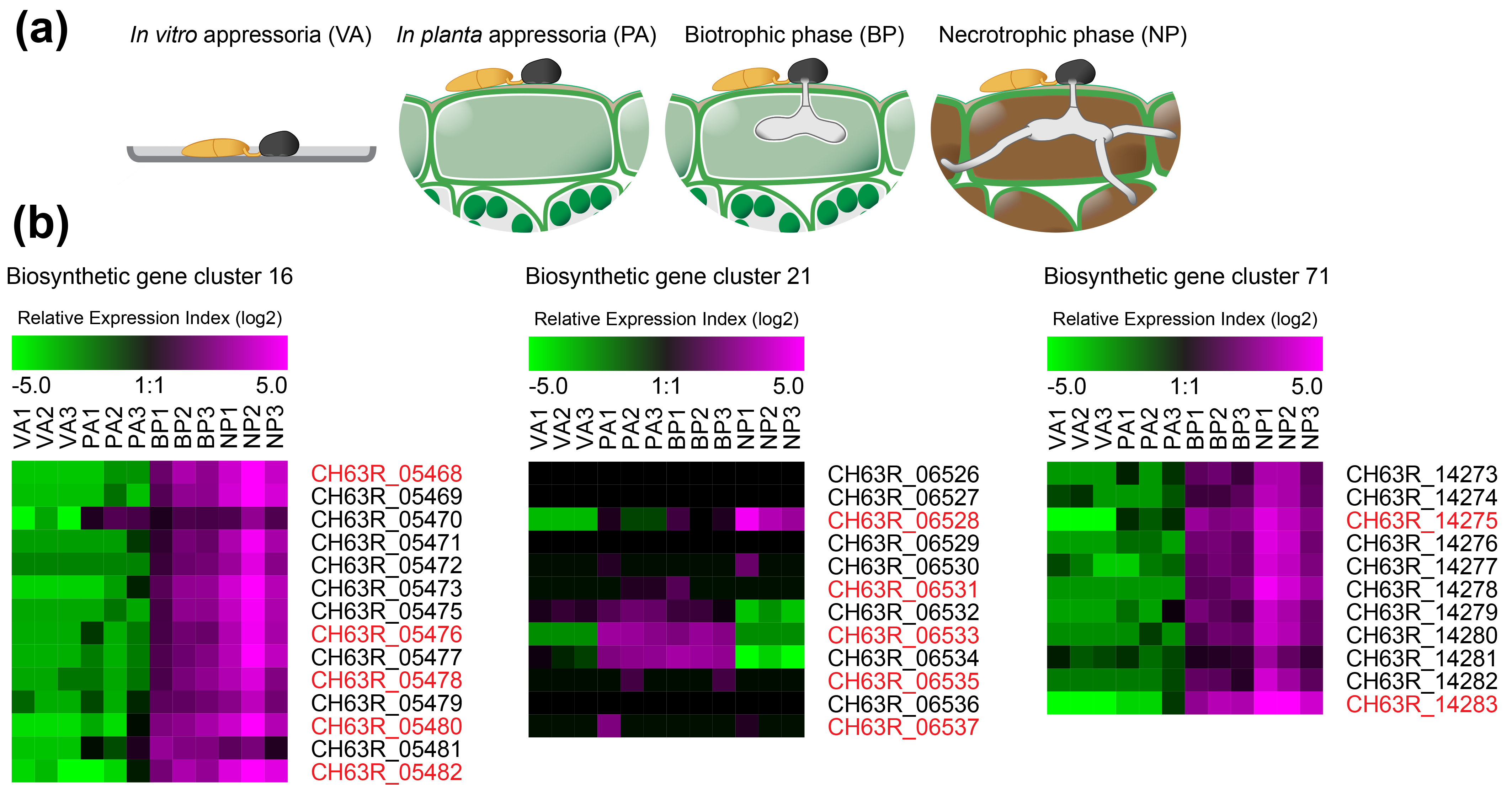

Supplement: Supplementary file 4 — Figure S4: Expression profiles of three C. higginsianum biosynthetic gene clusters (BGCs). (a) Schematic representation of the biological samples used in a transcriptomic study of in vitro formed appressoria (VA), in planta formed appressoria (PA), biotrophic phase (BP) and necrotrophic phase of Arabidopsis thaliana infection (O'Connell et al., 2012; Dallery et al., 2017). (b) Heatmap representations of the expression profiles of BGCs 16, 21 and 71. Under‐represented transcripts and over‐represented transcripts are shown as log2 Relative Expression Index (REI) in green and magenta, respectively. REI was computed as described by Hacquard et al. (2016). Gene IDs in red correspond to those proteins identified in C. higginsianum EVs. [file JEV2-11-e12216-s002.jpg]

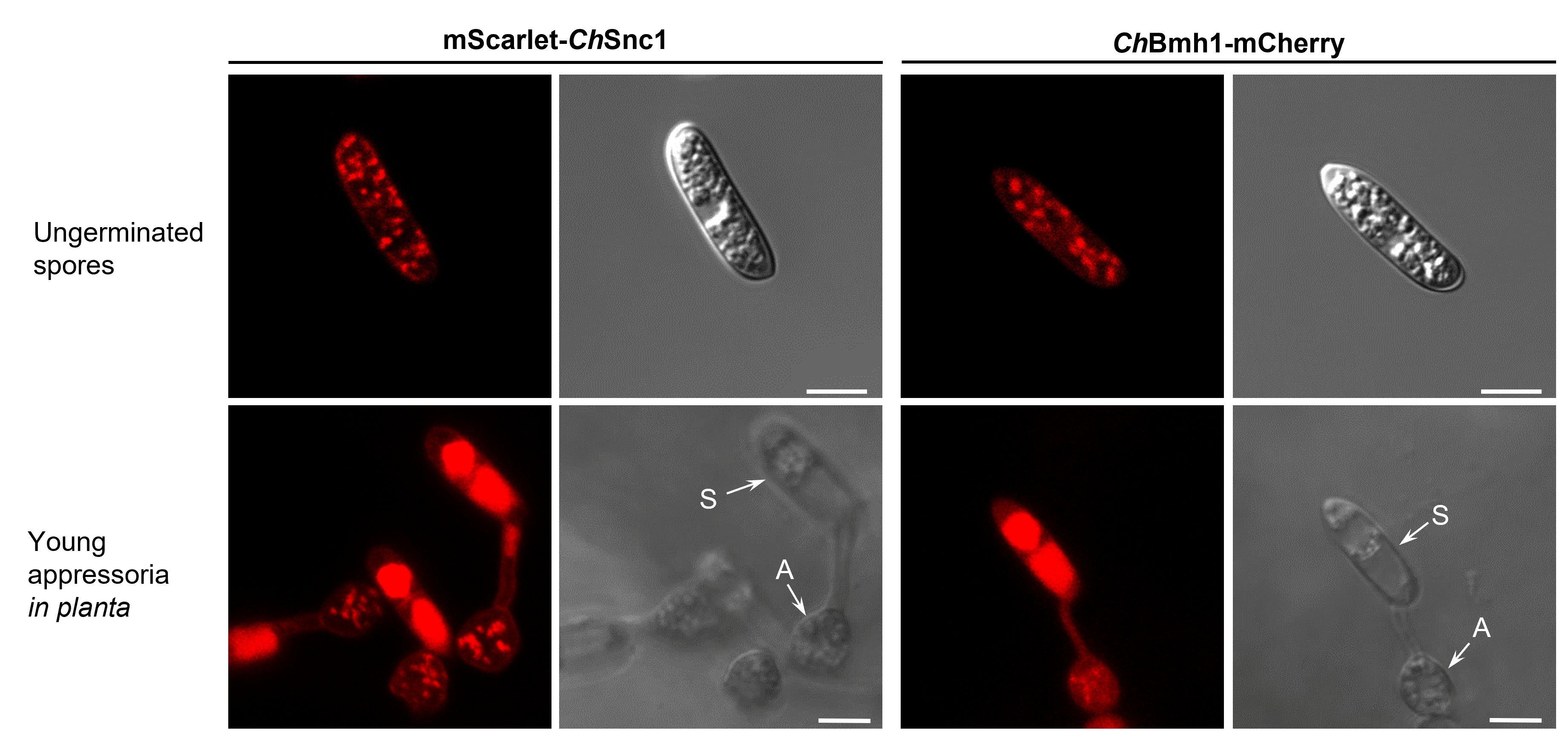

Supplement: Supplementary file 5 — FIGURE S5: Localization of EV marker proteins in spores and young appressoria in planta. Confocal laser scanning microscope images showing ungerminated spores of C. higginsianum and germinated spores forming young appressoria (arrowheads) on the surface of Arabidopsis cotyledons (7 h post‐inoculation), expressing either mScarlet‐ChSnc1 or ChBmh1‐mCherry. In ungerminated spores, both fluorescent markers localize to abundant punctate bodies in the cytoplasm. Following germination on the plant surface, both markers label large vacuoles inside germinated spores and small fluorescent puncta in the cytoplasm of appressoria, while mScarlet‐ChSnc1 also labelled the appressorium plasma membrane. Fluorescence and DIC channels are presented. Scale bars = 5 μm. [file JEV2-11-e12216-s005.tif]

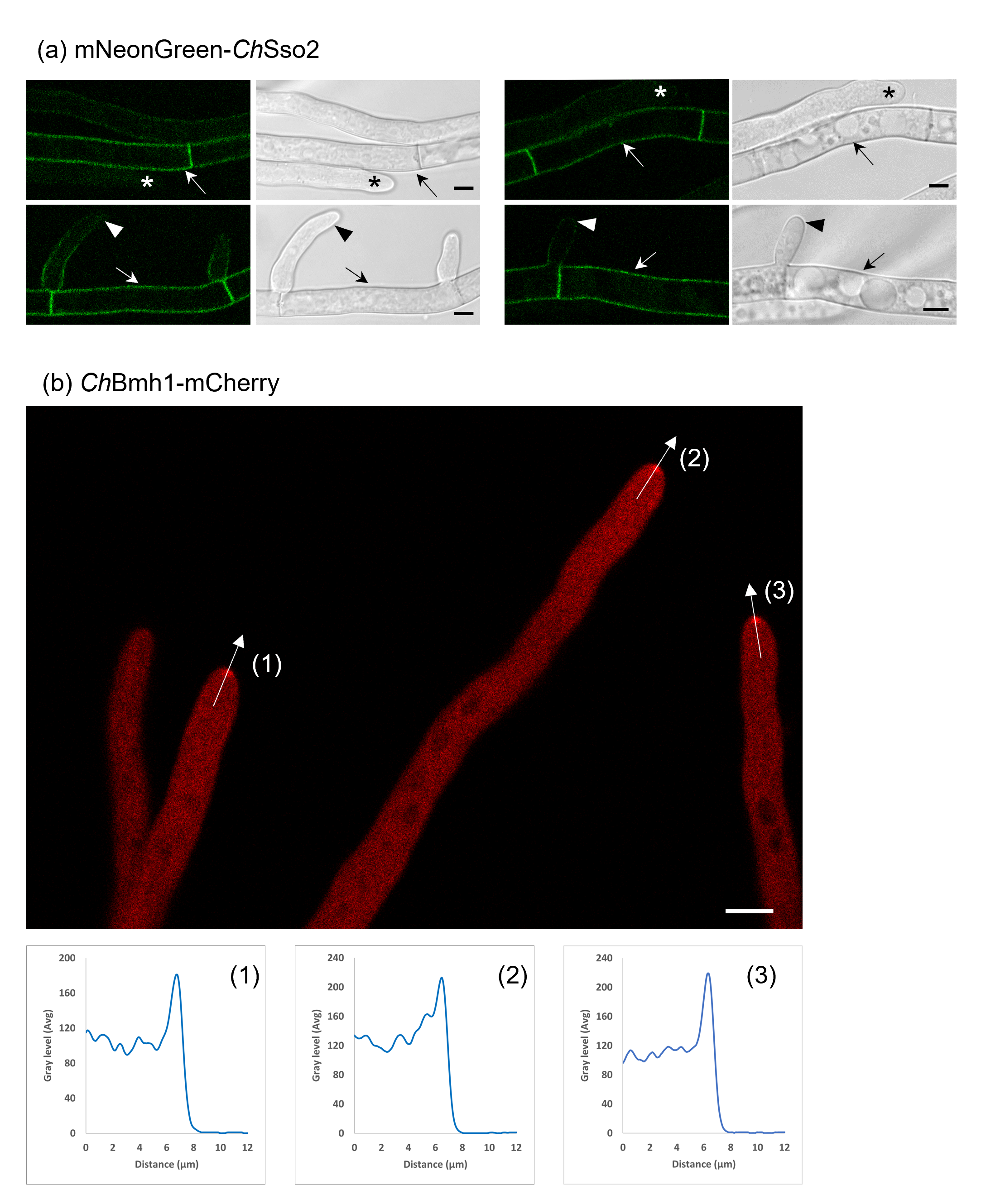

Supplement: Supplementary file 6 — Figure S6: Localization of EV marker proteins in vegetative hyphae of C. higginsianum growing on Mathur's agar medium. (a) Confocal single optical sections and corresponding bright‐field images showing mNeonGreen‐ChSso2 labelled the plasma membrane more strongly in older, septate hyphal compartments (arrows) compared to the apices of young hyphae (asterisks) and lateral branch hyphae (arrowheads). Scale bars = 5 μm. (b) Confocal single optical section showing hyphal apices expressing ChBmh1‐mCherry. A small focal concentration of the fluorescent marker at the apices of three different hyphae (1‐3) is consistent with labelling of the Spitzenkörper. The corresponding line scan fluorescence intensity plots confirm the stronger labelling of this apical region. Scale bar = 5 μm. [file JEV2-11-e12216-s011.tif]
