# Supplementary material for: The development of extracellular vesicle markers for the fungal phytopathogen Colletotrichum higginsianum
Source: J Extracell Vesicles. 2022 May 6;11(5):e12216. doi: 10.1002/jev2.12216 (PMC9077143; doi:10.1002/jev2.12216)
Supplement: Supplementary file 11 — TABLE S5: Best blastp hits from MIBiG database of BGCs linked to their biosynthetic product for the BGC21 of Colletotrichum higginsianum. TABLE S6: Best blastp hits from MIBiG database of BGCs linked to their biosynthetic product for the BGC71 of Colletotrichum higginsianum. TABLE S7: List of primers used for cloning [file JEV2-11-e12216-s008.docx]

**TABLE S5:** **Best blastp hits from MIBiG database of BGCs linked to their biosynthetic product for the BGC21 of *Colletotrichum higginsianum*.**

| **Protein_ID** | **Putative function^a^** | **Best hit MIBig v2.0^b^** | **e-value** | **Identity (%)** | **Coverage (%)** | **Organism** | **Linked molecule^c^** |
| --- | --- | --- | --- | --- | --- | --- | --- |
| OBR03047.1 | MFS Transporter | BGC0001449 | 6.21e-134 | 42.5 | 89.2 | *Thermothelomyces thermophilus* | UCS1025A |
| OBR03048.1 | Phosphotransferase | – | – | – | – | – | – |
| OBR03049.1 | Adenylation domain containing protein | – | – | – | – | – | – |
| OBR03050.1 | UstYa-like oxidase | – | – | – | – | – | – |
| OBR03051.1 | Enoyl reductase | BGC0000039 | 1.32e-162 | 61.0 | 100 | *Penicillium citrinum* | Compactin |
| OBR03052.1 | Esterase | BGC0000039 | 3.23e-60 | 42.5 | 94.8 | *Penicillium citrinum* | Compactin |
| OBR03053.1 | Dioxygenase | – | – | – | – | – | – |
| OBR03054.1 | UstYa-like oxidase | – | – | – | – | – | – |
| OBR03055.1 | Short-chain dehydrogenase/reductase | – | – | – | – | – | – |
| OBR03056.1 | MFS Transporter | – | – | – | – | – | – |
| OBR03057.1 | Polyketide synthase^§^ | BGC0000098 | 0 | 51.2 | 99.8 | *Monascus pilosus* | Monacolin K |

^a^ Based upon InterProScan results (InterPro release 86.0, 3^rd^ June 2021).

^b^ Kautsar et al., 2020

^c^ All the best blastp hits were found to be involved in the biosynthesis of a variety of biologically active molecules.

– denotes blastp results below the thresholds used in this study (less than 30% identity, more than 1.00e-50 e-value)

**TABLE S6:** **Best blastp hits from MIBiG database of BGCs linked to their biosynthetic product for the BGC71 of *Colletotrichum higginsianum*.**

| **Protein_ID** | **Putative function^a^** | **Best hit MIBig v2.0^b^** | **e-value** | **Identity (%)** | **Coverage (%)** | **Organism** | **Linked molecule^c^** |
| --- | --- | --- | --- | --- | --- | --- | --- |
| OBR03047.1 | MFS Transporter | BGC0001449 | 6.21E-134 | 42.5 | 89.2 | *Thermothelomyces thermophilus* | UCS1025A |
| OBR03048.1 | Phosphotransferase | – | – | – | – | – | – |
| OBR03049.1 | Adenylation domain containing protein | – | – | – | – | – | – |
| OBR03050.1 | UstYa-like oxidase | – | – | – | – | – | – |
| OBR03051.1 | Enoyl reductase | BGC0000039 | 1.32E-162 | 61.0 | 100 | *Penicillium citrinum* | Compactin |
| OBR03052.1 | Esterase | BGC0000039 | 3.23E-60 | 42.5 | 94.8 | *Penicillium citrinum* | Compactin |
| OBR03053.1 | Dioxygenase | – | – | – | – | – | – |
| OBR03054.1 | UstYa-like oxidase | – | – | – | – | – | – |
| OBR03055.1 | Short-chain dehydrogenase/reductase | – | – | – | – | – | – |
| OBR03056.1 | MFS Transporter | – | – | – | – | – | – |
| OBR03057.1 | Polyketide synthase^§^ | BGC0000098 | 0 | 51.2 | 99.8 | *Monascus pilosus* | Monacolin K |

^a^ Based upon InterProScan results (InterPro release 86.0, 3^rd^ June 2021).

^b^ Kautsar et al., 2020

^c^ Compactin and monacolin K are statins, a family of molecules inhibiting hydroxymethylglutaryl-CoA reductase enzyme and also with antifungal activities. The orthologous and complete BGC for statins in *C. higginsianum* is BGC44 (Dallery et al., 2017) but BGC71 shows also good homology with the statins cluster for three genes.

^§^The phylogenetic analysis of the KS domain of OBR03057.1 clearly shows it was originally a hybrid PKS-NRPS that has lost most of its NRPS domains during evolution. The protein is still predicted to be functional but as a polyketide synthase.

– denotes blastp results below the thresholds used in this study (less than 30% identity, more than 1.00e-50 e-value)

**TABLE S7: List of primers used for cloning**

| **Oligo Name** | **Sequence (5' - 3')** |
| --- | --- |
| prom_Snc1_F | gataagcttgatatcgaattcctgcagcccCATGCCGGCGAAAGCTCT |
| prom_Snc1_R | cttgatcactgcctcgcccttgctcaccatAGTCGCTGTGGTTACGGAACA |
| mScarlet_F | ATGGTGAGCAAGGGCGAGGCA |
| mScarlet_noSTOP_R | CTTGTACAGCTCGTCCATGCCG |
| Snc1_ORF+term_F | tccaccggcggcatggacgagctgtacaagATGCCCGAGGACGCCCC |
| Snc1_ORF+term_R | agtcacgttatgacctctagtggatcccccATCATGCGCCCTCCTGTTCT |
| prom_actin_F | taatgtactgaattaacgccgaattTCGGTCGTCGCCTTGC |
| prom_actin_R | tatcctcctcgcccttgctcaccatTTTGAATTAGGGTGGTGGGTGAG |
| mNeonGreen_F | ATGGTGAGCAAGGGCGAG |
| mNeonGreen_noSTOP_R | CTTGTACAGCTCGTCCATGCCCA |
| Sso2_ORF+term_F | gatgtgatgggcatggacgagctgtacaagATGTCGTACCAACAGTATAATC |
| Sso2_ORF+term_R | cgccgggcccaacatggtggcctaggaattGCCGAATGAATGATTTGGTC |
| prom_actin_F | gcttgatatcgaattcctgcagcccTCGGTCGTCGCCTTGCCTC |
| prom_actin_R | atcgccagacatTTTGAATTAGGGTGGTGGGTGAG |
| Bmh1_F | accctaattcaaaaTGTCTGGCGATGTAAGTACC |
| Bmh1_noSTOP_R | cttgctcaccatggatcccccgggaccGGAGGCGGCAGGAGTCTC |
| linker_mcherry_F | tcctgccgcctccggtcccgggggatccATGGTGAGCAAGGGCGAG |
| linker_mcherry_R | tggagctattaaaTTACTTGTACAGCTCGTCCATG |
| TrpC term_F | ctgtacaagtaaTTTAATAGCTCCATGTCAACAAG |
| TrpC term_R | cgttatgacctctagtggatcccccCGTCTAGAAAGAAGGATTACCTC |
